# Sulforaphane-enriched extracts from glucoraphanin-rich broccoli exert antimicrobial activity against gut pathogens in vitro and innovative cooking methods increase in vivo intestinal delivery of sulforaphane

**DOI:** 10.1007/s00394-020-02322-0

**Published:** 2020-07-10

**Authors:** Salah Abukhabta, Sameer Khalil Ghawi, Kimon Andreas Karatzas, Dimitris Charalampopoulos, Gordon McDougall, J. Will Allwood, Susan Verrall, Siobhan Lavery, Cheryl Latimer, L. Kirsty Pourshahidi, Roger Lawther, Gloria O’Connor, Ian Rowland, Chris I. R. Gill

**Affiliations:** 1 Department of Food and Nutritional Sciences, University of Reading, Whiteknights, P. O. Box 226, Reading, RC6 6AP UK; 2 Environmental and Biochemical Sciences Group, The James Hutton Institute, Invergowrie, Dundee, DD2 5DA Scotland, UK; 3 Nutrition Innovation Centre for Food and Health, School of Biomedical Sciences, University of Ulster, Cromore Road, Coleraine, BT52 1SA N. Ireland UK; 4 Altnagelvin Area Hospital, Western Health and Social Care Trust, Glenshane Road, Londonderry, UK

**Keywords:** Bioavailability, Antimicrobial, Beneforté, Sulforaphane, Glucoraphanin, Ileostomy

## Abstract

**Purpose:**

Studies on broccoli (*Brassica oleracea var. italica*) indicate beneficial effects against a range of chronic diseases, commonly attributed to their bioactive phytochemicals. Sulforaphane, the bioactive form of glucoraphanin, is formed by the action of the indigenous enzyme myrosinase. This study explored the role that digestion and cooking practices play in bioactivity and bioavailability, especially the rarely considered dose delivered to the colon.

**Methods:**

The antimicrobial activity of sulforaphane extracts from raw, cooked broccoli and cooked broccoli plus mustard seeds (as a source myrosinase) was assessed. The persistence of broccoli phytochemicals in the upper gastrointestinal tract was analysed in the ileal fluid of 11 ileostomates fed, in a cross-over design, broccoli soup prepared with and without mustard seeds.

**Results:**

The raw broccoli had no antimicrobial activity, except against *Bacillus cereus*, but cooked broccoli (with and without mustard seeds) showed considerable antimicrobial activity against various tested pathogens. The recovery of sulforaphane in ileal fluids post soup consumption was < 1% but the addition of mustard seeds increased colon-available sulforaphane sixfold. However, when sulforaphane was extracted from the ileal fluid with the highest sulforaphane content and tested against *Escherichia coli* K12, no inhibitory effects were observed. Analysis of glucosinolates composition in ileal fluids revealed noticeable inter-individual differences, with six “responding” participants showing increases in glucosinolates after broccoli soup consumption.

**Conclusions:**

Sulforaphane-rich broccoli extracts caused potent antimicrobial effects in vitro, and the consumption of sulforaphane-enriched broccoli soup may inhibit bacterial growth in the stomach and upper small intestine, but not in the terminal ileum or the colon.

**Electronic supplementary material:**

The online version of this article (10.1007/s00394-020-02322-0) contains supplementary material, which is available to authorized users.

## Introduction

Broccoli (*Brassica oleracea var. italica*) is a member of the Brassicaceae family of plants. Epidemiological studies, dietary trials, animal and in vitro investigations have revealed a range of potential beneficial effects of broccoli on a range of chronic diseases such as different types of cancers [1–4] and cardiovascular diseases [5–7]. The beneficial effects are commonly attributed to bioactive phytochemicals present that includes hydroxycinnamic acids, flavonoids (e.g., quercetin, kaempferol, myricetin), carotenoids (lutein) and glucosinolates (e.g., glucoraphanin) [8–10].

Glucosinolates are of great interest; in broccoli glucoraphanin is present in the highest quantity, comprising about 80% of the total, with smaller amounts of glucobrassicin, 4-methoxyglucobrassicin and 1-methoxyglucobrassicin also being found [11]. The biologically active forms of the glucosinolates are isothiocyanates which are generated by the action of the enzyme myrosinase that is released when the plant tissue is chopped or chewed [12]. In the case of glucoraphanin, the isothiocyanate formed is sulforaphane, less bioactive nitriles can also be formed by the activity of epithiospecifier protein (ESP) [13]. Sulforaphane has been shown in vitro to exert bioactivity [14–18] and to exert antibacterial effects against a range of food-borne pathogens and enteropathogenic microbes including *Listeria monocytogene*s, *Escherichia coli, Salmonella* Typhimurium and *Helicobacter pylori* [9, 19–21].

The effect of cooking *Brassica* vegetables on the absorption of isothiocyanates has been well studied [12]. It was reported that only 3.4% of sulforaphane was recovered in blood and urine after consuming microwaved broccoli due to myrosinase inactivation [22]. Similarly, isothiocyanates levels were three-fold higher in urine after consumption of raw broccoli compared with steamed broccoli (32.3% vs 10.2%) [23]. Fahey et al. [24] also demonstrated that active myrosinase was critical to the bioavailability of glucoraphanin in broccoli extracts as sulforaphane was 3–4 times more bioavailable in extracts with an active myrosinase enzyme than in extracts without. Similarly, Ghawi et al. [25] reported that the addition of mustard seeds, which contain a more resilient isoform of myrosinase, to heat-processed broccoli increased the formation of sulforaphane by 3–5fold.

In terms of bioavailability, Johnson [26] reported that isothiocyanates were absorbed through the small bowel and colon, where their metabolites were detected 2–3 h post consumption, with Petri and colleagues in an elegant study showing high levels of absorption for sulforaphane and quercetin 3,4'-diglucoside from an onion and broccoli extract by enterocytes using a perfused jejunal segment and determined that a proportion was effluxed back into the lumen as sulforaphane-glutathione and quercetin-3'-glucuronide [27]. An often-neglected aspect, however, is the role that digestion and cooking practices play in bioactivity and bioavailability, especially in relation to amounts of phytochemicals delivered to the colon.

In the present study, we have used ‘Beneforté broccoli’, a new variety that contains enhanced levels of glucoraphanin [28], which previous studies have demonstrated a threefold greater increase in the concentration of plasma sulforaphane compared to standard broccoli [29]. The aim of this study was two-fold: firstly to establish the antimicrobial activity of sulforaphane extracts from raw, cooked broccoli and cooked broccoli with a mustard seed (to provide myrosinase activity), and secondly to determine the persistence of broccoli phytochemicals and their metabolites in the upper gastrointestinal tract by analyzing the ileal output from ileostomates fed with a broccoli soup prepared with or without mustard seeds.

## Materials and methods

### Broccoli powder preparation and thermal processing

Beneforté broccoli was supplied by Staples Vegetables Ltd. (Boston, Lincolnshire, UK); mustard seeds (MS) were sourced from a local supermarket. Broccoli heads were cut (around 4–5 cm from top) and combined thoroughly. For the cooking experiments, 50 g portions were vacuum-packed in flexible polyethylene bags with dimensions of 24 × 24 cm, to stop glucoraphanin leaching into the processing water. Using a thermostatic water bath, broccoli was cooked at 100 °C for 12 min. Mustard seeds were ground and added as a powder. All samples of raw and cooked broccoli were frozen in dry ice before storage at −80 °C; following this, samples were lyophilized (VirTis SP Scientific, UK), ground using a coffee grinder and sieved (30 mesh). Samples were finally stored at −20 °C until further analysis. The solids content of fresh broccoli was 11.9% (w/w).

### Preparation of broccoli soup for the intervention study

The broccoli powder was prepared in the pilot plant facilities at the Department of Food and Nutritional Sciences at the University of Reading. All other ingredients were purchased from local stores at reading, uk. soup preparation was conducted at the pilot plant facilities. Good manufacturing practices (GMP) and hazard analysis critical control point (HACCP) were implemented throughout the preparation process to ensure the safety of the products. Ingredients to prepare the soup for the intervention study were shipped to Ulster University. Soup was locally prepared fresh prior to consumption. In brief, ingredients (Supplementary material 1), except broccoli powder and mustard seed powder, were dissolved in 50 ml cold water, and then added to 11 g broccoli powder in 150 ml boiling water. The resulting soup was then left to boil for 3 additional min to inactivate ESP [13]. The soup was left to cool down to 60 °C, and then mustard seed powder was added. The soup was allowed to stand for 10 min before it was served. Thirty-six untrained panellists were recruited to participate in a consumer evaluation test to determine the soup palatability at different broccoli powder concentrations (Supplementary material 2).

### Extraction and analysis of glucoraphanin from broccoli powder

Extraction of glucoraphanin from the broccoli samples was conducted as described previously by Oerlemans et al. [30] with minor modifications. Glucotropaeolin (0.25 ml of 1 mg/ml) (Santa Cruz Biotechnology, Heidelberg, Germany) was used as an internal standard. Freeze-dried samples (0.2 g) were extracted three times with 70% methanol at 70 °C for 10 min; these conditions were selected to inactivate myrosinase and avoid hydrolysis of the glucosinolates. Samples were centrifuged (3500*g*, 5 min), supernatants were combined and filtered (0.45 µm syringe filter) and finally topped up to 10 ml using 70% methanol. The extract was then purified using a mini column (HiTrap DEAE FF anion exchange column, 1 ml) (GE Healthcare, Chalfont, UK). Briefly, the column was washed five times with deionised water (1 ml each), and then 1 ml of the crude extract was passed through the column. The column was washed from unbound compounds with deionized water (2 × 1 ml) and sodium acetate buffer (2 × 1 ml; 20 mM, pH 5.0). Sulfatase enzyme (75 µl, 35 mg/ml) (Type H-1 from Helix pomatia, Sigma, Poole, UK) was added, and the column was incubated overnight at room temperature. The desulfo-glucosinolates were then eluted from the column using deionised water (4 × 1 ml). The eluate was dried using a rotary evaporator (70 °C) and re-dissolved in 0.5 ml of deionised water. The purified extract was filtered (0.45 µm syringe filter) and injected onto the HPLC. The desulfo-glucosinolates analysis was conducted with an HPLC–UV system (Agilent 1200, Manchester, UK) using an ACE 5 C18 reverse-phase column (150 × 4.6 mm) (Hichrom) with a flow rate of 1 ml/ min; absorbance measurement was conducted at a wavelength of 229 nm. The mobile phase was (A) water and (B) 20% acetonitrile. The following gradient was used: 100% (A) and 0% (B) for 1 min, B increased up to 100% over 20 min, returning to 100% (A) over 5 min and holding at 100% (A) for 4 min. The column temperature was set at 30 °C and the injection volume was 10 µl. Glucoraphanin quantification was carried out using desulfo-glucoraphanin as an external standard. All glucosinolate standards were sourced from Santa Cruz Biotechnology, Heidelberg, Germany. Organic solvents (HPLC-grade) were from Fisher Scientific (Loughborough, UK).

### Extraction and GC–MS analysis of sulforaphane and sulforaphane nitrile from broccoli powder

Extraction of sulforaphane and its nitrile analogue (sulforaphane nitrile) from broccoli samples was conducted as described by Ghawi et al. [25] with some modifications. 0.5 g of lyophilised broccoli powder was placed in a tube with 10 ml deionized water and incubated for 1 h at 30 °C. These conditions were selected to ensure complete hydrolysis of glucosinolates by myrosinase. Samples were centrifuged (5000*g*, 5 min) and the supernatant was collected; this step was repeated twice, and the supernatants were combined. To extract isothiocyanates and the nitrile analogues from the supernatant, 10 ml of dichloromethane were added to 10 ml of supernatant in a glass tube, vortexed for 1 min and centrifuged (5000*g* for 5 min); then the organic phase was collected. The extraction process was repeated two more times and the extracts were combined. About 3 g of unhydrated sodium sulphate were added to the extract to remove any excess water. The extract was then dried using a rotary evaporator (30 °C) and the eluate was re-dissolved in 0.7 ml dichloromethane and filtered using 0.22 µm filter.

The extracts were analyzed using GC–MS for sulforaphane and sulforaphane nitrile. GC–MS was performed using an Agilent 7850/5975 GC–MS system (Agilent, UK). The samples (1 µl) were injected onto a ZB-AAA capillary column (Phenomenex, USA) (0.25 µm film thickness, 15 m × 0.25 mm i.d.). The injection temperature was 250 °C and the split mode 1:20. The oven temperature was programmed from 110 to 320 °C at a rate of 30 °C/min. The flow rate of the helium carrier gas was 1.1 ml/min and the transfer line temperature held at 320 °C. Mass spectra were obtained by electron ionisation at 70 eV, and the mass scan was from 35 to 500 amu. Quantification of sulforaphane and sulforaphane nitrile was based on sulforaphane external standard.

### Extraction and HPLC analysis of sulforaphane from the soup and ileal fluids

Sulforaphane was extracted from broccoli soup based on the methodology described by Ghawi et al. [25]. One ml of the soup was centrifuged at 5000*g*, and the supernatant was collected; the pellet was extracted twice using 10 ml distilled water, and the supernatants were combined. Sulforaphane was extracted from the supernatant using 10 ml of dichloromethane for 1 min; the extraction step was carried out twice. The organic layers were collected (about 15 ml) and ~ 4 g sodium phosphate was added to remove any remaining water. Dichloromethane was evaporated using a rotary evaporator, and the pellet was dissolved in 0.6 ml of acetonitrile and filtered using a 0.45 µm filter.

Sulforaphane was extracted from ileal fluid samples as follows: 15 ml of the ileal fluid was centrifuged (14,500*g*, 10 min, 10 °C) and the supernatant was collected. The pellet was re-extracted with 10 ml water, and the supernatants were then combined. Sulforaphane was extracted from the supernatant using dichloromethane and treated as described above.

Ten microlitres of the filtrate were injected in an HPLC–UV system (Agilent 1200, Manchester, UK) using an ACE 5 C18 reverse-phase column (150 × 4.6 mm) (Hichrom) with a flow rate of 1 ml/min. The mobile phase was (A) water and (B) 100% acetonitrile. The following gradient was used: 100% (A) and 0% (B) for 1 min, B increased up to 100% over 20 min, returning to 100% (A) over 5 min and holding at 100% (A) for 4 min. The column temperature was set at 30 °C, whereas the absorbance was measured at 254 nm. Serial dilutions of sulforaphane standards (10–1600 µg/ml) were used to construct the standard curve. Organic solvents (HPLC-grade) were from Fisher Scientific (Loughborough, UK).

For comparison of sulforaphane concentrations by both GC and LC methods, the mean of each data set was used for statistical analysis. The Shapiro–Wilk test was used to test for normality and the Mann Whitney *U* test was used to compare groups. Significance was accepted at *p* < 0.05 unless otherwise specified. Analysis was carried out using SPSS (version 25 for Windows).

### Assessment of antimicrobial activity of sulforaphane extracts from broccoli powder

The bacteria used to test the antimicrobial activity of broccoli sulforaphane extracts included strains of the genera *Salmonella*, *Escherichia*, *Staphylococcus, Listeria* and *Bacillus*. Information on each strain and its origin is provided in Supplementary material 3. To understand the impact of broccoli sulforaphane extracts on food or in the gut, all bacterial strains with the exception of *E. coli* K12 used in these experiments are gram-positive and -negative foodborne pathogens. *E. coli* K12 is the most commonly used strain in microbiological research [31] and its use facilitated further genetic research we conducted on the antibacterial mode of action of broccoli sulforaphane extracts. We also included the pathogenic *E. coli* O157:H7-VT strain which although it is unable to produce verotoxin, it shares all other features with pathogenic strains. To get an insight on the antimicrobial mechanism of broccoli sulforaphane extracts we also included two *S.* Typhimurium strains, one with an intact and the other with defective RpoS. RpoS is the main stress gene regulator in *Salmonella enterica* and the majority of gram-negative bacteria [32]. We also included a pathogenic *L. monocytogenes* strain originally isolated from the human disease [33] with its isogenic mutant in glutamate decarboxylase *gadD2* that plays a role in antimicrobial resistance [34]. All bacterial strains were stored in 30% glycerol at −80 °C. The cells were cultured on nutrient agar (Oxoid) for up to 2 days, then three single colonies were obtained and transferred into 2 ml of phosphate buffer saline (PBS) solution. The turbidity of the suspensions was adjusted to match approximately a 0.5 McFarland standard (Fisher Scientific). The antimicrobial activity was tested using the disc diffusion method as described by Aires et al. [19]. In brief, from a bacterial suspension, a sterile cotton swab was spread onto Petri dishes (90 mm diameter) containing 20 ml of Mueller–Hinton Agar (Oxoid). Sterile filter paper discs (6 mm diameter; Oxoid) were impregnated with 100 µl of extracted sulforaphane samples and then placed on the agar plate; the plates were incubated in an inverted position overnight at 37 °C. The equivalent volume of solvent (acetonitrile) without sulforaphane extract was used as a negative control. Antibiotics, namely chloramphenicol (30 µg/ml), ampicillin (10 µg/ml), gentamicin (10 µg/ml) and tetracycline (30 µg/ml) (Oxoid) were used as positive controls. After overnight incubation, the diameter of the inhibitory zones around the disc was measured and recorded. All tests were performed in triplicate and the antibacterial activity was expressed as the mean of the diameters (in mm) of the inhibition zones. *T* test was used to compare the means of antibacterial activity, means were considered statistically different if *p* < 0.05 (Excel, Microsoft Office 365 ProPlus).

To determine the lowest concentration of sulforaphane needed to exert an antimicrobial effect, the minimum inhibition concentration (MIC) assay was conducted in a 96-well plate, as described by Wiegand et al. [35] using *E. coli* K12 as the indicator microorganism. Briefly, the 96 well plate was loaded with 150 µl of nutrient broth inoculated with 10^7^ CFU/ml of an overnight *E. coli* K12 culture (cell concentration of overnight culture ~ 10^9^ CFU/ml). Immediately after the addition of the culture, 150 µl of 2 mg/ml sulforaphane (LKT Laboratories, USA) were added in the first well, and then serial dilutions were made to achieve a range 1–0.03 mg/ml of sulforaphane. The plate was incubated at 37 °C in a micro-plate reader for 24 h and the optical density measured at 620 nm and plotted over time. The experiment was performed in triplicate for each dilution.

### Dietary intervention study with ileostomy patients

The use of ileostomy patients combined with metabolomics can provide insight into the processes of biotransformation and absorption in the upper gastrointestinal tract that impact the phytochemicals in the ingested plant material, in the case of broccoli glucoraphanin and sulforaphane, potentially influencing bioactivity and health benefits [36, 37]. The ileostomy feeding study, an acute randomised, single-blind, placebo-controlled crossover study with 1-week washout, used a soup prepared (described earlier) from Beneforté broccoli, a hybrid of *Brassica oleracea* and *Brassica villosa* [28], supplied by Staples Vegetables Ltd (Boston, Lincolnshire, UK) and mustard seeds were sourced from a local supermarket (ASDA, Reading, UK). Soup was chosen as the intervention food product to ensure that the participants received a homogenous and uniform food providing a consistent quantity of broccoli phytochemicals delivered in the form of a powdered broccoli-based soup. Thereby minimising the impact of any interplant composition variation and ensuring that effects observed biologically were a consequence of inter-person variation in ADME alone. As no studies existed on broccoli consumption and ileostomists, a 4-h post-consumption sampling point selected to ensure capture of the appearance of phytochemicals from the intervention food. This choice was consistent with ileostomy studies that used a liquid-based vehicle to deliver phytochemicals and showed substantive quantities to be evident in ileal fluid after 4 h [38, 39]. While a one week washout period for the study was considered appropriate as it ensured that broccoli phytochemicals would have passed through the gastrointestinal tract after consumption of a single bolus meal (soup, 200 ml).

Ethical approval (14/SC/1326) for the study was received from the South Central-Hampshire Research Ethics Committee and Ulster University. All participants gave written informed consent and the study was conducted in accordance with the Helsinki Declaration. Participants were recruited between Jan 2015 and Feb 2015 from clinics at Altnagelvin Hospital. The intervention study ran between March 2015 and April 2015. The study was registered at clinicaltrials.gov as NCT04113928. The study was conducted in 11 ileostomists (7 males, 4 females) age range 32–63 years, who had undergone terminal ileostomies and were at least 1.5 years post-operative prior to the study and were non-smokers.

After obtaining consent, participants were randomly assigned, in blocks of four using a random-number generator (www.randomization.com), to either the intervention or the control. In total, 12 participants were randomised to 2 groups. One participant subsequently withdrew from the study prior to sampling. Participants were asked to follow a restriction diet, avoiding dark green leafy vegetable and mustard-like vegetables especially broccoli, cabbage, Brussels sprouts, watercress, rocket, spinach, onions, spring onions, radish, horseradish for 48 h before each clinic visit. Following an overnight fast, the participants provided an ileal fluid sample (T 0 h) then consumed 200 ml of freshly prepared broccoli soup (described above) with/without the addition of mustard seed, within 15 min, a second ileal fluid sample was collected 4 h post consumption (T 4 h). The ileal fluid samples were collected and processed as described in McDougall et al. [40]. Weights and pH of the ileal fluid were recorded, before dilution with ice-cold distilled water as required, dependent on viscosity, before being homogenised in a chilled Waring blender for 30 s, and storage of aliquots at −80 °C in preparation for subsequent analysis.

### Targeted analysis of glucosinolates in ileal fluids

Ileal fluids were extracted using the procedure outlined previously [40]. Briefly, frozen ileal fluid samples were thawed and vortex-mixed, and duplicate 2.0 ± 0.1 g samples were weighed into 15 ml centrifuge tubes. These were extracted using 3 ml of ultrapure water containing aqueous 0.1% formic acid and 20 mM diethyl dithiocarbamate (DDC). The tubes were vortex-mixed for 3 × 30 s, then sonicated in a water bath for 1 min. All procedures were carried out at 5 °C. After centrifugation (2500*g*, 10 min, 5 °C), the supernatants were transferred to new tubes. The pellets were extracted twice using 3 ml of 0.1% formic acid in methanol containing 20 mM DDC, and the supernatants combined and vortex-mixed. A subsample of 4 mL was removed and dried in a Speed-Vac. The dried samples were resuspended in 0.5 mL of 10% (v/v) acetonitrile containing 500 µM morin as an internal standard then transferred into filter vials (Thomson, 0.45 μM PTFE filter vial: Bioprocess Engineering Services Ltd., Ashford, Kent, UK). The samples were analysed by liquid chromatography-mass spectrometry (LC–MS) using an Agilent 1260 Infinity HPLC 6230 time of flight (TOF) mass spectrometry system operated under MassHunter (Agilent, Ltd. UK) software. The TOF/MS system was tuned and calibrated according to the manufacturers recommended procedures for use across 80–2000 m*/*z in negative mode. Sub 3 ppm mass accuracies were achieved by applying the recommended reference mass locking solution to a secondary ESI probe via a secondary HPLC pump at a flow rate of 2.5 mL/min with 1:100 splitter (25 μL/min to source) using the *m/z* values at 112.985587 and 1033.988109 in the negative mode as standard reference locks. The method for quantification of Gls is given in the Supplementary material 4.

### Data handling and statistical analysis for non-targeted analysis

The LC–MS raw data were converted into MZML centroid format using the Proteowizard MSConvert software package (https://proteowizard.sourceforge.net/). The data was then deconvoluted using the XCMS online package (https://xcmsonline.scripps.edu/) producing a Microsoft Excel-based XY matrix of the paired RT and *m/z* of each feature against the peak intensity for each sample. The dataset was next subjected to automated peak annotation workflows within PutMedID [41, 42] which grouped *m/z* features likely associated with the same compound (i.e., a ‘peak group’). Accurate mass differences between *m/z* within each peak group were next calculated to allow the annotation of the parent *m/z*, isotope and adduct ions, as well as common in-source fragments. The neutral accurate mass was then matched to libraries of possible metabolites (Plant Metabolic Network PlantCyc database (https://www.plantcyc.org/ and the Manchester Metabolomics Database (MMD: https://dbkgroup.org/MMD/). After annotation, non-grouped low intensity features, redundant isotope, adduct and fragment features were removed from the dataset to reduce data complexity. Principal components analysis and optimized partial least squares-discriminant analysis was applied to the XCMS data to identify components that increased in the after broccoli ileal fluids. Full details are given in Supplementary material 5.

### Antimicrobial activity of sulforaphane extracted from ileostomy fluids against *Escherichia coli* K12

To assess the antimicrobial activity, sulforaphane was extracted from ileostomy fluid in a similar way to that from soup, with the exception that the final pellet was dissolved in 0.6 ml nutrient broth (Oxoid) instead of acetonitrile. The concentration of sulforaphane in nutrient broth was 3.14 µM. A 96-well plate titration method was used to assess the inhibitory effect of extracted sulforaphane against *E. coli* K12 cells. The sulforaphane containing nutrient broth was filtered through a 0.20 µm filter, and 200 µl were dropped into each well. *E. coli* K12 grown overnight in nutrient broth at 37 °C was used as inoculum. Each well was inoculated with an impregnate needle, and the plates were incubated at 37 °C for 24 h; the absorbance was measured at 620 nm using a plate reader (Sunrise™, Tecan, Austria). The obtained growth curves were compared with that of the control (no sulforaphane addition) in terms of their lag phase, growth curve pattern and final optical density to evaluate the effect of antimicrobial effect of sulforaphane.

## Results

### Quantification of glucoraphanin, sulforaphane and sulforaphane nitrile in broccoli samples

**Fig. 1:**
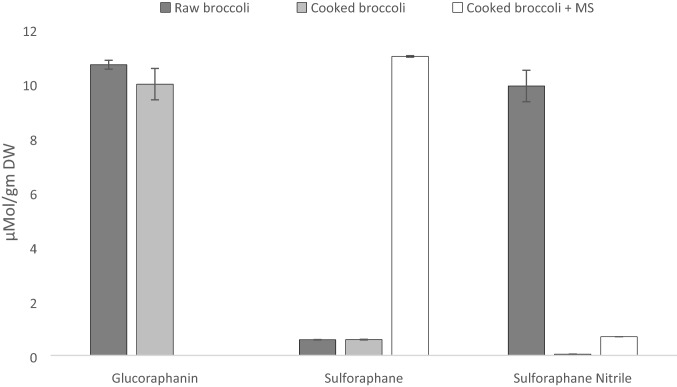
Glucoraphanin, sulforaphane and sulforaphane nitrile contents in raw, cooked (100 °C for 12 min) broccoli, and cooked broccoli after the addition of mustard seed powder (MS)

The glucoraphanin content in raw broccoli was 10.68 µmol/g broccoli powder and cooking did not significantly reduce content (9.97 µmol/g) (Fig. 1). Sulforaphane was low in raw broccoli (0.57 µmol/g DW) with significantly higher levels of sulforaphane nitrile (9.9 µmol/g DW). Cooking of broccoli did not increase the concentration of sulforaphane (0.58 µmol/g DW) compared to raw (Fig. 1), probably as cooking inactivated the endogenous myrosinase activity. However, the addition of mustard seed powder (2%) to cooked broccoli significantly (*P* < 0.05) increased sulforaphane formation from 0.58 to 10.90 µmol/g DW. Although sulforaphane nitrile level increased after the addition of mustard seeds (0.05 vs 0.69 μmol/g DW), this increase is low compared to the level formed in broccoli before cooking (0.69 vs 9.9 µmol/g DW).

### Sulforaphane content in broccoli soups

Following the initial studies, a broccoli soup was prepared which contained 26.5 µmol of sulforaphane per 200 ml portion (0.13 µmol/ml soup). The addition of 2% mustard seed powder at the cooling stage of the soup preparation process (~ 60 °C) increased the level of sulforaphane by nearly fourfold in the soup, 102 µmol per 200 ml portion (0.51 µmol/ml soup).

### Antimicrobial activities of broccoli sulforaphane extracts

The raw broccoli extracts showed no antimicrobial activity (Table 1), except against *B. cereus*. In contrast, the extracts from cooked broccoli (with and without mustard seeds) showed considerable antimicrobial activity against the tested strains. Furthermore, the extracts from cooked broccoli with added mustard seed exhibited significantly higher activity (*T *test; *p* < 0.05) than the cooked broccoli without MS. This increased activity could be interpreted as being due to the increased levels of isothiocyanate, mainly sulforaphane (Fig. 1), formed from glucoraphanin (~ 81% of total glucosinolate content in broccoli [11]), presumably due to the activity of myrosinase from the MS. Other compounds such as allyl isothiocyanate originating from MS may contribute to the antimicrobial activity, however, this contribution is likely to be minor as MS was added in very small quantity to broccoli (2%). The greatest zone of inhibition was reported for *B. cereus* (13.7–46.7 mm). Lower antimicrobial activities were generally observed for the gram-positive bacteria such as *S. aureus* and *L. monocytogenes* strains compared to the gram-negative ones such as *S. enterica,* and *E. coli*.

**Table 1:** Inhibition zones (in mm) from disc diffusion method assessing the antimicrobial activity of sulforaphane-rich dichloromethane extracts from raw, cooked and cooked (plus mustard seed) broccoli and of different antibiotics against various bacterial strains

Bacterial stain	Raw broccoli	Cooked broccoli	Cooked broccoli plus mustard seed	Chloram-phenicol (30 µg/ml)	Ampicillin (10 µg/ml)	Gentamicin (10 µg/ml)	Tetracycline (30 µg/ml)
	Mean (mm) ± SD, *n* = 3						
*S.* Typhimurium DT104 str. 10	ND	15.3 ± 0.6 *	23.3 ± 0.6 **	ND	0.0 ± 0.0	18.0 ± 0.0	9.0 ± 0.0
*S.* Typhimurium DT104 str.30	ND	15.3 ± 0.6 *	22.3 ± 0.6 **	29.0 ± 0.0	26.5 ± 0.7	18.5 ± 0.7	27.5 ± 0.7
*S.* Hadar	ND	15.0 ± 1.0 *	20.7 ± 0.6 **	27.0 ± 0.0	26.5 ± 0.7	17.0 ± 0.0	ND
*S.* Virchow	ND	15.7 ± 0.6 *	25.0 ± 0.0 **	27.5 ± 0.7	27.5 ± 0.7	19.0 ± 0.0	28.0 ± 0.0
*S.* Heidelberg	ND	14.7 ± 0.6 *	21.7 ± 0.6 **	26.5 ± 0.7	26.0 ± 0	19.0 ± 0.0	27.5 ± 0.7
*S.* Anatum	ND	15.3 ± 0.6 *	23.0 ± 0.0 **	24.0 ± 1.4	21.0 ± 0	16.5 ± 0.7	25.5 ± 0.7
*E. coli* K12	ND	14.7 ± 0.6 *	23.3 ± 0.6 **	29.5 ± 0.7	ND	22.0 ± 0.0	31.0 ± 0.0
*E. coli* O157:H7-VT	ND	14.3 ± 0.6 *	20.3 ± 0.6 **	30.0 ± 0.0	ND	20.5 ± 0.7	30.5 ± 0.7
*S. aureus* 408	ND	12.7 ± 0.6 *	18.3 ± 0.6 **	35.0 ± 0.0	24.5 ± 0.7	32.0 ± 0.0	42.5 ± 0.7
*B. cereus* 138	19 ± 1.0	34.7 ± 0.6 *	45.3 ± 0.6 **	NA	NA	NA	NA
*L.* monocytogenes 10403S WT	ND	10.3 ± 0.6 *	18.3 ± 0.6 **	25.0 ± 0.0	21.5 ± 0.7	23.0 ± 0	31.5 ± 0.7
*L. monocytogenes* 10403S ∆*gadD2*	ND	10.3 ± 0.6 *	18.0 ± 0.0 **	25.5 ± 0.7	22.0 ± 0	25.0 ± 0	32.5 ± 0.7

*Indicates a statistically significant difference (*p* < 0.05) between inhibition zones with raw broccoli and cooked broccoli. **Indicates a statistically significant difference (*p* < 0.05) between inhibition zones with cooked broccoli and cooked broccoli with mustard seeds. Statistically significant difference was assessed with the use of *T* test

Interestingly, all cooked broccoli extracts demonstrated a strong inhibitory effect against both the antibiotic-resistant S. Typhimurium str.10 and str.30 (inhibition zones ranging from 10 to 25 mm; Table 1). With *E. coli and S.* Typhimurium strains, the cooked broccoli with MS extracts were as effective as gentamycin. The cooked broccoli extracts also demonstrated an inhibitory effect on *L. monocytogenes* (inhibition zones ranging from 10 to 18 mm). The minimum inhibitory concentration (MIC, the concentration at which cell growth was completely inhibited) for pure sulforaphane against *E. coli* K12 was established at 1 mg/ml (5.65 mM), (Supplementary material 6), with concentrations as low as 0.03 mg/ml exerting some inhibition of bacterial growth.

### Ileostomy feeding study with broccoli soup

**Fig. 2:**
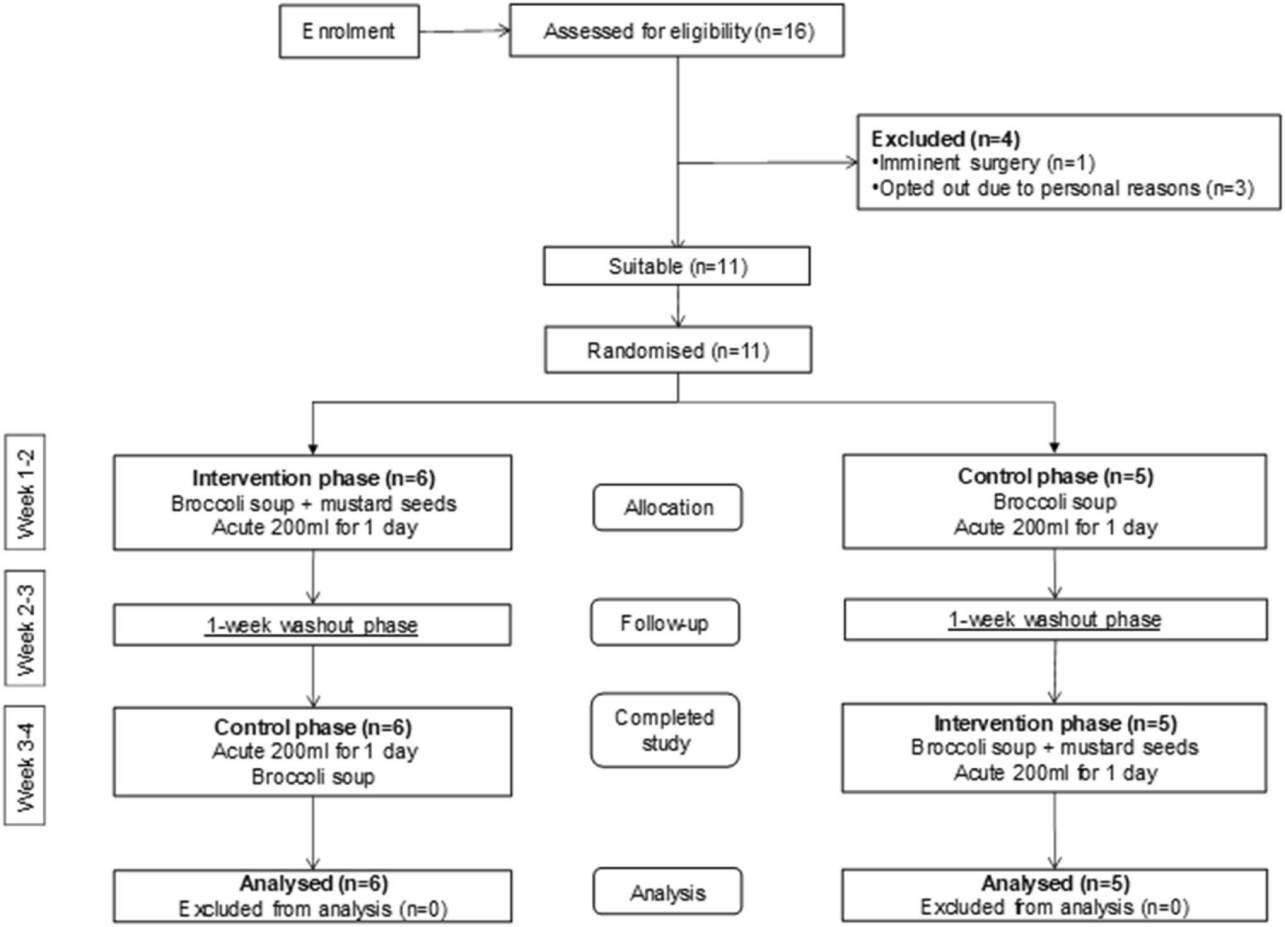
Consort diagram, progress of participants through the intervention study

Eleven ileostomates (males *n* = 7, females *n* = 4) were enrolled on this acute randomised, single-blind, placebo-controlled two-way crossover trial. The intervention was conducted as per the protocol and with the consent of participants, all 11 subjects completed the study and there were no adverse events associated with the consumption of broccoli soup (Fig. 2). The study population had a mean group age of 53.3. ± 9.2 years. The average weight of ileal fluid day 1 (0 h) 233 g ± 84, pH 5.9 ± 0.7, day 2 214 g ± 84, pH 5.9 ± 0.6.

The group mean content of sulforaphane in the ileal fluids from subjects given broccoli without mustard seeds was 0.17 µmol, with considerable inter-individual variation (Table 2). This represents a recovery of < 1% of the applied sulforaphane in the original soup. The addition of MS to the broccoli soup significantly increased the group mean amount of sulforaphane in the ileal fluids to 1.05 µmol (a six-fold increase), once again with a substantial inter-individual variation. This is a significant increase in colonic availability of sulforaphane.

**Table 2:** Sulforaphane content in all ileal fluids post consumption (4 h)

	Broccoli soup without mustard seeds		Broccoli soup with mustard seeds	
	Sulforaphane (SF) per soup portion (200 ml) SF = 26 ± 0.01 µmol		Sulforaphane (SF) per soup portion (200 ml) SF = 102.2 ± 0.03 µmol	
Subject no	Total SF (µmol)	Recovery (%)	Total SF (µmol)	Recovery (%)
S01	0.00	0.00	0.49	0.48
s02	0.00	0.00	3.21	3.15
s04	0.36	1.38	3.14	3.08
s05	0.48	1.83	0.00	0.00
S06	0.26	1.01	0.26	0.26
S07	0.00	0.00	1.88	1.84
s08	0.26	1.00	0.42	0.41
S09	0.33	1.26	0.66	0.65
S10	0.00	0.00	0.10	0.10
s11	0.07	0.27	0.94	0.92
s12	0.11	0.42	0.49	0.48
Group mean	0.17 ± 0.17	0.65	1.05* ± 0.66	1.03

Data as group means (*n* = 11) were assessed for normality and compared by Mann Whitney *U* test, *p = 0.007

**Fig. 3:**
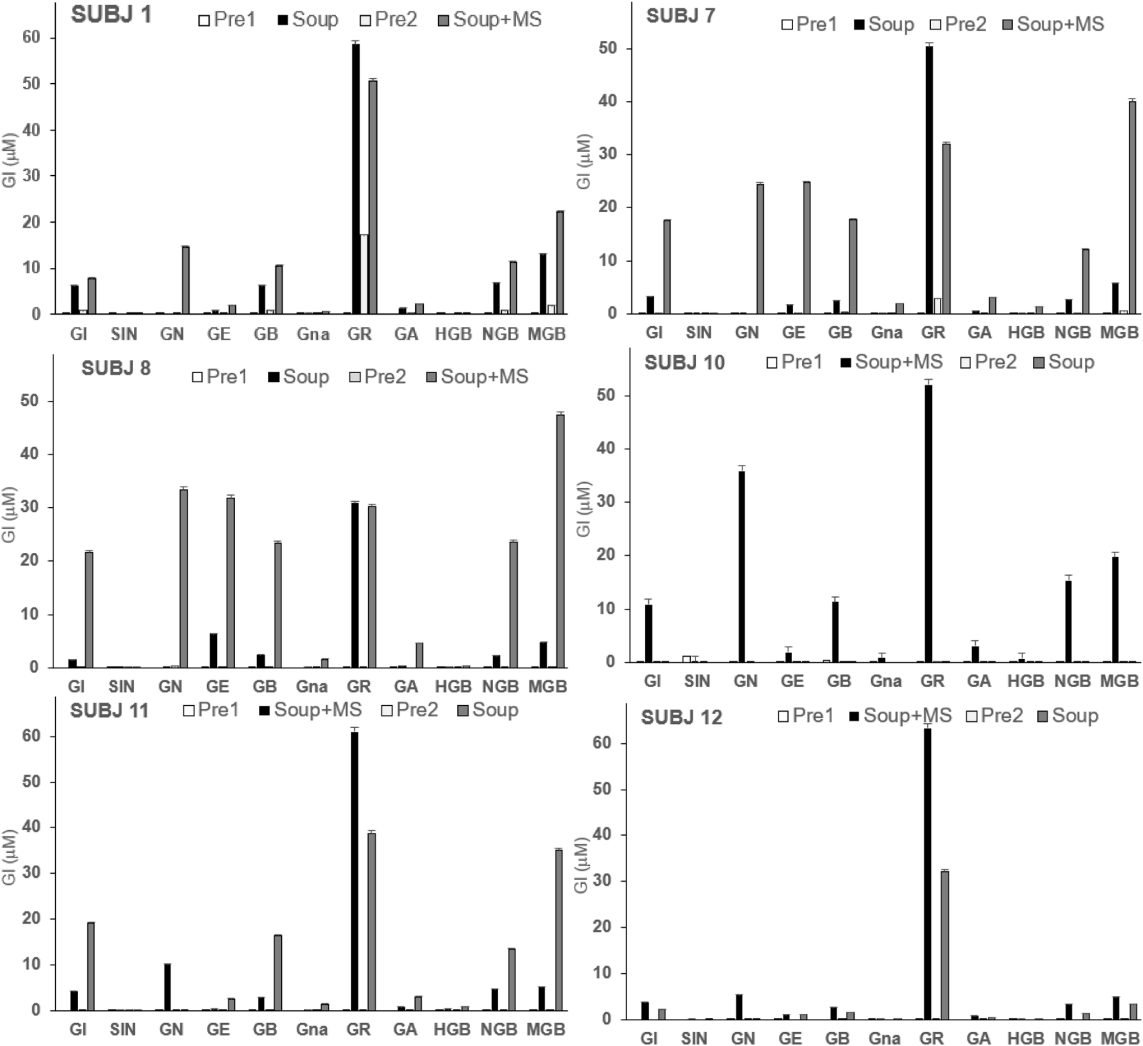
GIucosinolates contents in ileal samples before and after broccoli soups, subjects 1, 7, 8,10, 11, and 12. *GI* Glucoiberin, *SIN* sinigrin, *GN* gluconapin, *GE* glucoerucin, *GB* glucobrassicin, *Gna* gluconasturtiin, *GR* glucoraphanin, *GA* glucoalysin, *HGB* hydroxyglucobrassicin, *NGB* neoglucobrassicin, *MGB* neoglucobrassicin

Glucosinolates (GIs) content and composition were quantified in ileal fluids before and after broccoli soup intake using targeted LC–MS TOF analysis against individual glucosinolate standards. There were considerable inter-individual differences in response; only six of the eleven subjects (SUBJs 1, 7, 8, 10, 11 and 12) showed clear increases in GIs after soup intake (Fig. 3) and there was considerable variation in the composition and levels of GIs between the responders. The five “non-responding” subjects either had high levels of GIs in pre-intervention samples (SUBJs 2, 5 and 6) or showed very low levels of GIs in any samples (SUBJs 4 and 9). The GIs composition in the ileal fluids from the responders had glucoraphanin as the dominant component but there was considerable variation in the composition of other GIs between the responding subjects. In SUBJs 10 and 12, Gls content was more pronounced in the broccoli soup without mustard seeds. This is expected as myrosinase in broccoli was inactivated during soup preparation. However, the same pattern does not hold for the other responders.

In addition, a non-targeted approach was applied to identify components that also increased in ileal fluids following broccoli soup intake. The MS data were deconvoluted using XCMS software and multivariate statistical analyses (Principal Component and OPLS-DA analysis) were employed to identify components that increased after soup intake (Supplementary material 7, Figs. 1, 2, 3, 4). As expected, the non-targeted analysis of the responders also identified the presence of the same GIs in the ileal fluids (Supplementary material 8), confirming the targeted analysis. Allyl isothiocyanate (*m/z* 98; predicted formula C_4_H_5_NS) was also detected, which could arise from the mustard seeds as a breakdown product of sinigrin [43].

However, other components were putatively identified which most probably arise from the broccoli consumed. The phenolic compound, feruloyl-sinapoyl-gentiobiose (*m/z* 723; predicted formula C_33_H_40_O_18_), is characteristic of broccoli florets (https://foodb.ca/compounds/FDB002177) but can be found in other *Brassicas*. Kaempferol sophoroside (*m/z* 609, C_27_H_30_O_16_) was also identified and this is known to be a major phenolic component of broccoli (https://phenol-explorer.eu/contents/food/263). Feruloyl putrescine was also identified and this has been confirmed in other studies on broccoli [44]. A component with *m/z* 163 (predicted formula C_9_H_8_O_3_) was also identified which could be phenylpyruvate. The absence of other more abundant broccoli phenolics (as detailed in https://phenol-explorer.eu/contents/food/263) probably reflects their relative stability in the gut.

### Antimicrobial effect of sulforaphane extracted from ileostomy fluid against *E. coli* K12

Following analysis of the ileal samples (Fig. 2), subject S04 was identified as having the highest amounts of sulforaphane at 3.14 µmol (equivalent to 41 µM) and was selected as a candidate to assess inhibitory effect against *E. coli* K12. It was not possible to carry out anti-microbial assessment using the ileal fluid per se, given the inherent presence of small intestinal microbiota, consequently sulforaphane was extracted from the ileal fluid, dried, and re-constituted to the original ileal fluid concentration (41 µM) in nutrient broth to evaluate its antimicrobial activity against *E. coli* K12. The results indicated no difference in the growth of *E. coli* in the presence of sulforaphane compared to the control (data not shown). The MIC of pure sulforaphane against *E. coli* K12 was 5.65 mM (1 mg/ml) but 171 µM (0.03 mg/ml) exerted some inhibition of bacterial cell growth.

## Discussion

Studies on broccoli (*Brassica oleracea var*. *italica*) indicate beneficial effects against a range of chronic diseases, commonly attributed to their bioactive phytochemicals. Sulforaphane, the bioactive form of glucoraphanin, is formed by the action of the enzyme myrosinase. This study explored the role that digestion and cooking practices play in bioactivity and bioavailability, especially the rarely considered dose delivered to the colon.

The processing of the broccoli from raw to the cooked forms with and without the added enzyme myrosinase, in the form of mustard seeds, altered the phytochemical composition of the various broccoli powders (Fig. 1). Cooking broccoli did not significantly reduce the glucoraphanin content and indeed only limited glucosinolate thermal degradation has been noted at processing temperatures lower than 110 °C [30, 45]. Consequently, the cooking process likely denatured broccoli’s endogenous thermo-labile myrosinase preventing hydrolysis of the glucosinolates. Sulforaphane was low in raw broccoli but higher levels of sulforaphane nitrile were present, which agrees with previous literature [46–48]. The formation of sulforaphane nitrile rather than sulforaphane in raw broccoli is due to the action of epithiospecifier protein (ESP), a non-catalytic cofactor of myrosinase that promotes the formation of epithionitriles from alkenyl glucosinolates. The formation of sulforaphane nitrile was substantially reduced after cooking which indicates that ESP, the highly temperature-sensitive protein [45], was inactivated during cooking. The addition of mustard seeds powder (2%) to cooked broccoli soup significantly increased sulforaphane formation (fourfold) while sulforaphane nitrile was detected at low levels. Therefore, the addition of low concentrations of mustard seeds powder, which contains a thermally resistant myrosinase [45, 49]**,** to cooked broccoli has the potential to intensify sulforaphane formation.

The in vitro antimicrobial activities of broccoli sulforaphane extracts were in line with the results from previous studies which have shown that pure sulforaphane has a broad antimicrobial spectrum effect against both gram-negative and gram-positive bacteria [19, 20, 50–53]. However, the present study showed that gram-negative bacteria, such as *E. coli* and *S. enterica* were more susceptible to sulforaphane than gram-positive bacteria, such as *L. monocytogenes* and *S. aureus*, with the exception of the gram-positive *B. cereus*. In this study, we used two highly similar *S.* Typhimurium DT104 strains (strains 10 and 30). Strain 30 possesses a fully functional RpoS, whilst strain 10 possesses a defective RpoS due to an amber non-sense codon [32]. RpoS is the main stress gene regulator in *S. enterica* and other gram-negative bacteria involved in resistance to a variety of stresses [32]. Interestingly, both strains were equally susceptible to broccoli extracts suggesting that RpoS and its regulon that comprises hundreds of stress genes may not play a role in resistance against broccoli extracts or sulforaphane. We also included a mutant in the glutamate decarboxylase gene *gadD2* in *L. monocytogenes* which plays a role in antimicrobial resistance, did not show a different behaviour comparing to the wild type *E. coli* K12 suggesting that also this gene does not play a role in resistance to broccoli extracts or sulforaphane.

Bacteria, such as *E. coli* O157: H7, *S.* Typhimurium DT104 and *L. monocytogenes*, which were tested in this study are associated with gastrointestinal infections and food poisoning and can cause considerable health problems to humans. For instance, *E. coli* O157:H7 can cause acute haemorrhagic diarrhoea [54]. Enterotoxigenic *E. coli* can colonize the upper bowel and cause watery diarrhoea which in some cases can lead to death [55], and *S.* Typhimurium DT104 can also cause diarrhoea, fever, headache, nausea, abdominal pain and vomiting. *L. monocytogenes* is another important potentially pathogenic bacterium which can cause listeriosis through the colonisation of the gastrointestinal tract, which is one of the deadliest foodborne infections in the Developed World [56]. While the antimicrobial activity of the broccoli extracts was comparable to ampicillin, chloramphenicol, tetracycline and gentamycin (Table 1). It should be stated that this comparison is not based on weight since antibiotics are normally used in low quantities. However, this comparison is valid since the concentrations of antibiotics and broccoli used in these experiments are comparable to the levels that would be found in the intestinal tract following ingestion of broccoli and that in an infection site following administration of these antibiotics. Overall, the results obtained from this study, suggest that cooked broccoli extracts might be promising natural agents for controlling human pathogens during the gastrointestinal digestion of foods, with the addition of myrosinase potentiating the antimicrobial activity possibly due to the enhanced level of sulforaphane. Considered from the in vivo perspective, following consumption of the broccoli soup(s) the ileal recovery of sulforaphane was < 1% which indicates early absorption in the small intestine consistent with observations by Petri et al. [27] who reported ~ 74% absorption of sulforaphane in a perfused jejunum section and highlighted that a proportion was conjugated to sulforaphane-glutathione and excreted back into the lumen and, therefore, low colonic-availability. However, the addition of MS to the broccoli soup significantly increased the amount of sulforaphane available to the colon by sixfold, although there were considerable inter-individual differences in responses evident. Similarly, a study in healthy participants reported that consumption of broccoli (200 g) with brown mustard seeds increased urinary excretion of the sulforaphane metabolite sulforaphane *N*-acetyl-l-cysteine fourfold [57]. Sulforaphane extracted from the ileal fluid of subject S04 (which had the highest sulforaphane content) did not show antimicrobial activity against *E. coli* K12 presumably as the sulforaphane concentration was below the observed MIC (Supplementary material 6).

The targeted and non-targeted LC–MS approaches adopted in the study identified the presence of the expected GIs in the ileal fluids. Other compounds were also identified in the non-targeted approach including B-vitamins such as pantothenamide (*m/z* 217, C_9_H_18_N_2_O_4_) and metabolites of biotin (vitamin B7), norbiotin (*m/z* 229, C_9_H_14_N_2_O_3_S), bisnorbiotin (*m/z* 215, C_8_H_12_N_2_O_3_S) and biotin sulfoxide (*m/z* 259, C_10_H_16_N_2_O_4_S). Broccoli has a low content of biotin so these metabolites probably arise from the cheese and milk powder in the soup mix (Table in Supplementary material 1). Pantothenamide is a food additive used as a precursor for pantothenic acid (vitamin B5) and probably also arises from these other soup ingredients. There are many putative peptides in the post-soup ileal fluids. Whilst one of these (e.g., Leu-Leu; leucyl-leucine; *m/z* 243, C_12_H_24_N_2_O_3_) has been identified in broccoli previously [44], the others could arise from the digestion of proteins in the cheese and/or sauce powder used in the formulation of the soups. It is intriguing that these components from the more prosaic soup components are identified as soup-specific by the non-targeted LCMS approach.

Other components were putatively identified as drugs, which may be a consequence of the skewed nature of the available databases towards pharmaceuticals, but some components may be more relevant. For example, colestipol (*m/z* 302, C_11_H_28_ClN_5_O) is a bile acid sequestrant often prescribed for ileostomy patients (https://bnf.nice.org.uk/drug/colestipol-hydrochloride.html). Napsagatran (*m/z* 597; C_26_H_36_N_6_O_7_S) was putatively identified but it is unknown if this drug was prescribed to the subjects. We can only assume that these drugs have been taken with or before the soups and appear increased due to transit through the gut with the food bolus.

We also attempted to assess the antimicrobial effect of sulforaphane extracted from ileostomy fluid against *E. coli* K12, however, it was determined that even the broccoli soup modified to increase sulforaphane could not deliver sufficient concentrations to the terminal portion of the ileum to exert antimicrobial effects colonically. However, the higher concentration of sulforaphane in the broccoli soup with added myrosinase would be within the inhibitory range determined, so it is possible that this broccoli soup could inhibit the growth of bacteria present in the stomach and upper small intestine rather than the lower intestine. Also, the GIs present in the ileal fluids (Fig. 3) represent reservoirs of sulforaphane (i.e., from the major component, glucoraphanin, or other isothiocyanates from other GIs) that could be released by the colonic microflora [26]. Bacteria that could be potentially affected by sulforaphane include *Helicobacter pylori*, a pathogenic bacterium associated with gastric and duodenal ulcers, which has been suggested through human trials to be inhibited by sulforaphane [58–61].

To conclude, sulforaphane-enriched extracts from beneforté broccoli exerted anti-microbial activity against gut pathogens in vitro and the inclusion of a heat-tolerant source of myrosinase in cooked broccoli can increase intestinal delivery of sulforaphane, albeit to very low levels.

## Electronic supplementary material

Below is the link to the electronic supplementary material.

Supplementary file1 (DOCX 2175 kb)
