# Supplementary material for: Sulforaphane-enriched extracts from glucoraphanin-rich broccoli exert antimicrobial activity against gut pathogens in vitro and innovative cooking methods increase in vivo intestinal delivery of sulforaphane
Source: Eur J Nutr. 2020 Jul 10;60(3):1263–76. doi: 10.1007/s00394-020-02322-0 (PMC7987625; doi:10.1007/s00394-020-02322-0)
Supplement: Supplementary file 1 — Supplementary file1 (DOCX 2175 kb) [file 394_2020_2322_MOESM1_ESM.docx]

**Supplementary materials**

**Supplementary material 1** Ingredients used to prepare the broccoli soup

| **Ingredients** | **Intervention Soup** |
| --- | --- |
| Broccoli powder (g) | 11 |
| ^*^Sauce Mix (g) | 32.5 |
| ^**^Cheese powder (g) | 5 |
| Salt (g) | 0.3 |
| Mustard seeds powder (g) | 0.16 |
| Water (ml) | 200 |

^*^Sauce mix ingredients (Sainsbury's Cheddar Cheese Sauce Mix, Item code: 7444127): wheat flour, cheddar cheese powder (16%), cheese powder (16%), maize starch, skimmed milk powder, salt, yeast extract, flavouring, maltodextrin, whey powder, mustard powder, colours (curcumin, paprika extract).

^**^Cheese powder (Sainsbury's Shaved Parmesan Cheese, Item code: 1304473)

**Supplementary material 2** Consumer acceptance of soup

Consumer evaluation of broccoli soups: Thirty-six untrained panellists were recruited to participate in a consumer evaluation test to determine the soup palatability. The test was carried out at the Sensory Science Centre at the University of Reading using individual sensory booths. The sample soup was provided, the liking of the soup (overall liking followed by liking of appearance, taste and texture) was scored on a 9-point hedonic category scale (1: dislike extremely, 9: like extremely). The participants were also asked for their free text comments on soup.

|  | **Intervention Soup** |
| --- | --- |
| Overall liking | 6.7 ^a^ ±1.4 |
| Liking of appearance | 7.1 ^a^ ±0.9 |
| Liking of taste | 6.2 ^a^ ±1.7 |
| Liking of texture | 6.7 ^a^ ±1.1 |

The results show the mean values of consumers’ scores of overall liking, as well as liking of appearance, taste and texture of the broccoli soup. The soup was scored slightly to moderately liked giving an average score of ~ 7, which indicated their suitability for carrying out a dietary intervention study.

**Supplementary material 3** Bacteria used in this study to assess the inhibitory effect of sulforaphane extracted from broccoli samples

| **Strain** | **Details** | **Reference or source** |
| --- | --- | --- |
| *S*. Typhimurium DT 104 | Strain 10 Human, mutated defective *rpoS* gene | Jorgensen et al. (2000) |
| *S*. Typhimurium DT 104 | Strain 30 Bovine, intact *rpoS* gene | Jorgensen et al. (2000) |
| *S*. Hadar | P518721 Meat | PHE^1^, Microbiological Services |
| *S*. Virchow | P518634 Pet food | PHE, Microbiological Services |
| *S.* Heidelberg | S172457 Chicken | PHE, Microbiological Services |
| *S*. Anatum | S180332 Dried Food | PHE, Microbiological Services |
| *E. coli* K12 | KEIO collection | Baba et al. (2006) |
| *E. coli* O157:H7-VT | NCTC 12900, non VT | PHLS^2^ |
| *S. aureus* 408 | NCIMB 6571 | Heatley (1944) |
| *B. cereus* 138 | NCFB 578 Milk | Garvie and Stone (1953) |
| *L. monocytogenes* 10403S WT | Human skin lesion | Alonso III et al. (2011) |
| *L. monocytogenes* 10403S ∆*gadD2* | Human skin lesion, deleted *gadD2* gene | Feehily et al. (2014) |

^1^Now Public Health England, Microbiology Services

^2^Public Health Laboratory Service

Baba T, Ara T, Hasegawa M, et al (2006) Construction of Escherichia coli K-12 in-frame, single-gene knockout mutants: the Keio collection. Molecular systems biology *2*: 2006.0008. <https://doi.org/10.1038/msb4100050>

Heatley NG (1944) A method for the assay of penicillin. The Biochemical journal 38(1): 61-65. <https://doi.org/10.1042/bj0380061>

Garvie E, Stone M (1953) The associative growth of Bacillus cereus and Streptococcus lactis in milk. Journal of Dairy Research 20(1): 29-35. <https://doi:10.1017/S002202990000666X>

Alonzo F, Bobo LD, Skiest DJ, Freitag NE (2011) Evidence for subpopulations of Listeria monocytogenes with enhanced invasion of cardiac cells. Journal of medical microbiology, 60(4): 423-434. <https://doi.org/10.1099/jmm.0.027185-0>

Feehily C, Finnerty A, Casey PG, Hill C, Gahan CGM, et al (2014) Divergent Evolution of the Activity and Regulation of the Glutamate Decarboxylase Systems in Listeria monocytogenes EGD-e and 10403S: Roles in Virulence and Acid Tolerance. PLOS ONE 9(11): e112649. <https://doi.org/10.1371/journal.pone.0112649>

**Supplementary Material 4** Quantification of GIs in the ileal fluid

The samples were stored in the HPLC autosampler at 10 °C and an injection volume of 5 µl was applied. The HPLC was operated at a flow rate of 400 µl/min with a Kinetex C18 column (100 Ä, 150 x 4.6 mm, 2.6 µm particle size; Phenomenex Ltd. Macclesfield U.K) fitted with a corresponding guard column. The column was maintained at 40 °C. Solvent A was HPLC grade water and solvent B was HPLC grade acetonitrile (JT Baker Ltd, Scientific and Chemical Supplies Ltd, Aberdeen U.K.) both acidified with 0.1% [v/v] formic acid (Ultima mass spectrometry grade, Fisher Scientific Ltd. U.K.). The HPLC gradient programme was as follows: From 0-5 mins, the eluent was held at 5 % then from 5-25 min % B increased to 35% B, from 25-27 min increased from 35 to 75% B, held at 75% from 27-32 min, returned to 5% B from 32-33 min then re-equilibrated at 5% B from 33-38 min.

The HPLC column eluent was transferred to the Agilent 1260 Infinity PDA detector with spectra collected from 200-600 nm at a sample rate was 5 Hz. Additionally, three channels were collected at 280 nm, 365 nm and 520 nm. The PDA detector eluent was next transferred to the Agilent 6230 TOF/MS. From 0-2.5 mins, the eluent was sent to waste then directed to the ESI probe from 2.5-33 minutes before returning to waste from 33-38 mins during the column re-equilibration phase. The samples were analysed in triplicate in a completely randomised order, with QC samples and control blank samples applied to monitor instrument performance and sample carry over.

Stock solutions at 11 mg/mL were prepared for each of the reference standards (glucoiberin, glucoraphanin, glucoeirolin, progoitrin, sinigrin, sinalbin, gluconapin, glucotropaeolin, glucoerucin, glucobrassicin and gluconasturtiin; LGC Standards, Teddington, Middlesex, UK) in HPLC grade methanol. The stock solutions were mixed in equal volumes, generating a standard mix of glucosinolates (GIs) at 1 mg/mL. The standard mix was diluted with 20% HPLC grade methanol to 100 µM and then 50, 25, 10, 5, 2.5 and 1 µM. The solutions were then transferred to 2 mL HPLC vials before being subjected to the same LC-MS method as sample extracts from low to high concentration. GIs in ileal samples were identified by comparison against the available standard GIs and by using *m/z* values and predicted formula (all correct at < 3 ppm) for GIs reported to be present in broccoli**^*^**. The content of GIs in samples was quantified against standard curves for each identified GI and expressed as means of triplicate analyses ± standard errors. Other GIs in the samples were quantified against glucoiberin as the nearest structural equivalent GI.

**^*^** Sasaki K, Neyazaki M, Shindo K, Ogawa T, Momose M. Quantitative profiling of glucosinolates by LC-MS analysis reveals several cultivars of cabbage and kale as promising sources of sulforaphane. J Chromatogr B Analyt Technol Biomed Life Sci. 2012;903:171‐176. <https://doi.org/10.1016/j.jchromb.2012.07.017>

**Supplementary Material 5** Principal components analysis and optimized partial least squares-discriminant analysis

Principal component analysis (PCA) was applied to the XCMS data from all subjects (SIMCA-P 12.0.1.0 software) which showed some separation between before and after soup samples especially when a discriminant Optimized Partial Least Squares discriminant analysis (OPLS-DA) was applied (see Supplementary Material 7, Fig. 1). PCA analysis was carried out on the data from the responding subjects and there was a clear discrimination of the before and after samples (Fig. 2). OPLS-DA was performed on the responder data with two classifications, before feeding and after feeding, which produced a model with a Q2 score of 0.787 (Fig. 3). Using the loadings plot from the OPLS-DA (Fig. 4), a list of over 200 molecular features was extracted that most influenced the separation towards the after-soup state. These were filtered by removal of adducts then removal of features that only had one adduct but lacked more probable adducts. Overall 72 components were then putatively identified by their MS properties, possible molecular formulae generated in the PutMedID process and checked against literature (Supplementary Material 8). Some possible *m/z* values could be fitted to formula and others gave possible formula that could not be identified. It should be noted that the same loadings list could be generated from the loadings plot of the PCA of the responders i.e. subjects showing an increase in GI after broccoli consumption.

**Supplementary Material 6** Estimation of minimum inhibitory concentration (MIC) of pure sulforaphane against *E. coli* K12

**Supplementary Material 7**

**Fig. 1 OPLS-DA PCA plot of all subjects**

The PCA plot describes > 36 % of the variation and the Q2 score was 0.406. B-1 = before 1, A1 = after soup 1, B2 = before 2, A2 = after soup 2.

**B-1**

**A-1**

**B-2**

**A-2**

**Fig. 2 PCA plot of responders**

This plot describes % of the variation and had a Q2 score of 0.279.

**Fig. 3 OPLS-DA plot of responders**

This plot had a Q2 score of 0.787.

**B-1**

**A-1**

**B-2**

**A-2**

**Fig.4 Loadings plot from OPLS-DA of responders**


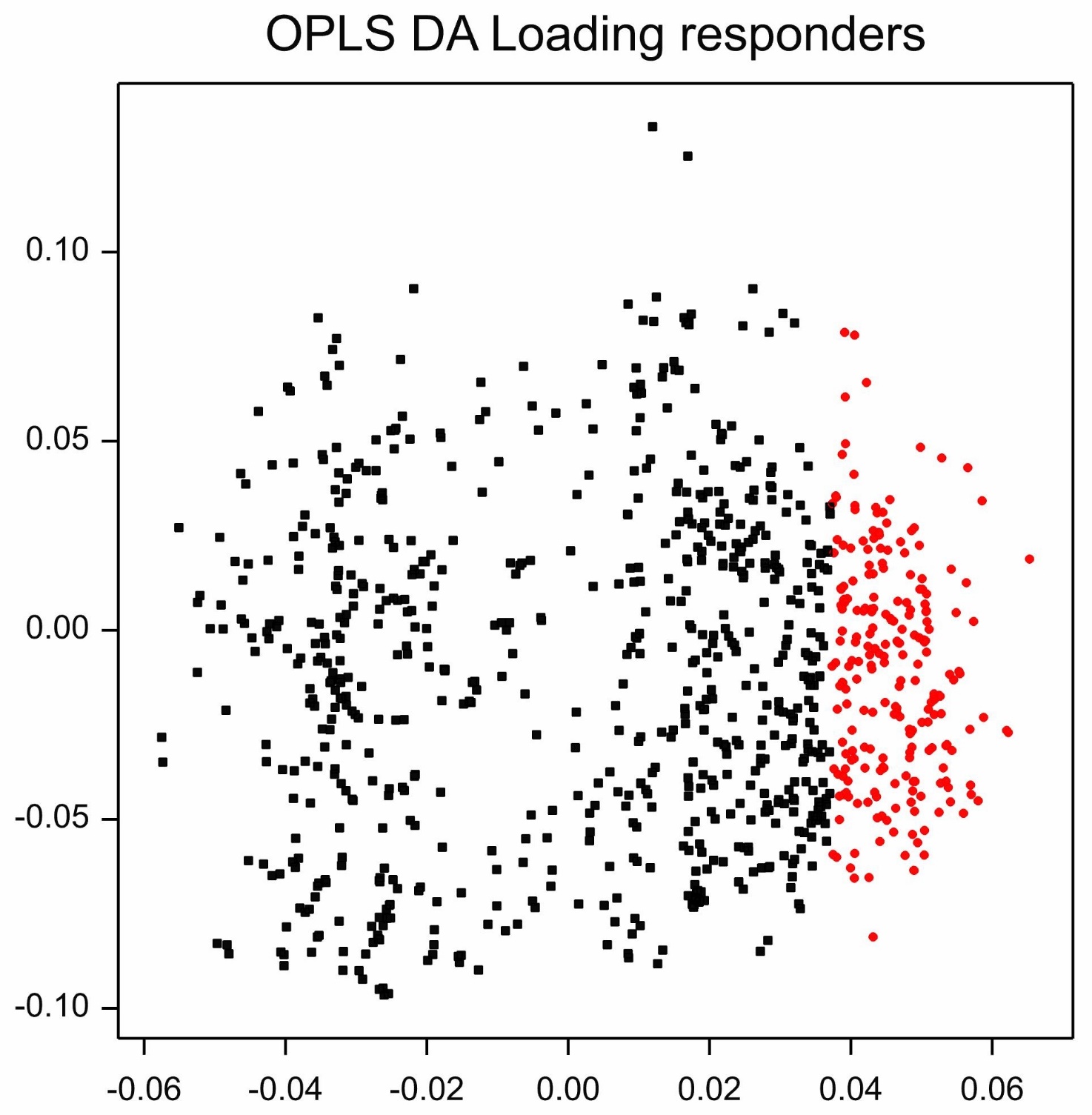


The *m/z* values in red (loadings value > 0.035) most influenced the separation between the after soup and before soup ileal samples and were selected for further study.

**Supplementary Material 8** Compounds putatively identified as increased in after Broccoli soup ileal fluid samples

|  | ***m/z* [M-H]^-^** | **RT (min)** | **Error (ppm)** | **Molecular Formulae** | **Potential Identity** |
| --- | --- | --- | --- | --- | --- |
| **1** | 254.9260 | 3.286 | 2.812 | NF | Unknown |
| **2** | 195.0510 | 3.442 | 0.183 | C_6_H_12_O_7_ | Carboxy-D-arabinitol or  D-Gluconic acid |
| **3** | 422.0260 | 3.729 | 2.946 | C_11_H_21_NO_10_S_3_ | Glucoiberin |
| **4** | 436.0420 | 3.924 | 4.231 | C_12_H_23_NO_10_S_3_ | Glucoraphanin |
| **5** | 604.0690 | 3.936 | 1.308 | C_16_H_25_N_5_O_16_P_2_ | GDP-mannose; GDP-glucose |
| **6** | 306.9620 | 3.948 | 4.804 | NF | Unknown |
| **7** | 304.1510 | 4.493 | 2.162 | C_12_H_25_NO_2_ | Unknown |
| **8** | 163.0400 | 4.539 | 0.54 | C_9_H_8_O_3_ | Phenylpyruvate |
| **9** | 779.2450 | 5.662 | 2.263 | C_29_H_46_N_6_O_9_S | Unknown |
| **10** | 352.0710 | 5.805 | 1.533 | C_10_H_22_N_4_O_2_S_2_ | Unknown |
| **11** | 395.1560 | 5.979 | 2.923 | C_15_H_26_N_4_O_7_ | Ala-Leu-Asp-Gly |
| **12** | 98.0071 | 6.913 | 0.865 | C_4_H_5_N_S_ | Allyl isothiocyanate |
| **13** | 217.1190 | 7.539 | 0.417 | C_9_H_18_N_2_O_4_ | Pantothenamide |
| **14** | 474.2200 | 7.72 | 0.393 | C_19_H_35_N_3_O_5_ | Unknown |
| **15** | 318.1670 | 8.205 | 1.869 | C_13_H_27_NO_2_ | Unknown |
| **16** | 244.1300 | 8.646 | 0.320 | C_10_H_16_N_2_O_4_ | Prolyl-hydroxyproline |
| **17** | 486.2570 | 8.748 | 3.984 | C_27_H_43_NO_2_ | Etioline; Leptinidine; Solasodine or Tomatidinol |
| **18** | 201.1250 | 9.11 | 4.537 | C_9_H_18_N_2_O_3_ | Leu-Ala |
| **19** | 231.1350 | 9.159 | 0.303 | C_10_H_20_N_2_O_4_ | Thr-Leu |
| **20** | 639.3820 | 9.412 | 4.378 | C_40_H_58_O_4_; C_41_H_56_O_3_; C_40_H_54_O | Unknown |
| **21** | 245.1140 | 9.552 | 0.845 | C_10_H_18_N_2_O_5_ | L-beta-Aspartyl-L-Leucine or  L-gamma-Glutamyl-L-Valine |
| **22** | 267.0960 | 9.552 | 1.790 | C_10_H_18_N_2_O_5_ | L-beta-Aspartyl-L-Leucine or  L-gamma-Glutamyl-L-Valine (Na) |
| **23** | 302.1720 | 10.27 | 3.901 | C_11_H_28_ClN_5_O | Colestipol |
| **24** | 517.2260 | 10.407 | 0.623 | C_21_H_38_N_4_O_8_ | Unknown |
| **25** | 229.1560 | 10.616 | 0.895 | C_11_H_19_NO_3_ | Unknown |
| **26** | 293.1500 | 11.071 | 1.126 | C_15_H_22_N_2_O_4_ | Unknown |
| **27** | 265.1190 | 11.195 | 0.996 | C_14_H_22_O_2_ | Unknown |
| **28** | 597.2120 | 11.478 | 1.908 | C_26_H_36_N_6_O_7_S | Napsagatran (Na) |
| **29** | 301.0800 | 11.489 | 0.366 | C_13_H_16_N_2_O_5_ | Asp-Phe |
| **30** | 399.2240 | 11.768 | 1.246 | C_18_H_29_N_3_O_6_ | *N*-(3-Propylcarbamoyloxirane-2-Carbonyl)-Isoleucyl-Proline |
| **31** | 523.2150 | 11.949 | 0.140 | C_28_H_40_O_2_ | Octacosaoctaenoic acid |
| **32** | 447.0540 | 12.107 | 0.564 | C_16_H_20_N_2_O_9_S_2_ | Glucobrassicin |
| **33** | 263.1400 | 12.76 | 0.324 | C_14_H_20_N_2_O_3_ | Feruloyl putrescine |
| **34** | 808.3730 | 13.148 | 3.953 | C_42_H_59_N_3_O_10_ | Unknown |
| **35** | 311.1580 | 13.307 | 1.073 | C_12_H_24_N_2_O_3_ | Leu-Leu (Na-FA adduct) |
| **36** | 293.1500 | 13.716 | 3.973 | C1_5_H_22_N_2_O_4_ | Tyr-Leu |
| **37** | 315.1320 | 13.716 | 2.275 | C_15_H_22_N_2_O_4_ | Tyr-Leu (Na) |
| **38** | 330.2030 | 14.099 | 1.237 | C_15_H_29_N_3_O_5_ | Ile-Thr-Val |
| **39** | 422.0580 | 14.107 | 2.229 | C_15_H_21_NO_9_S_2_ | Gluconasturtiin |
| **40** | 263.1400 | 14.111 | 0.289 | C_14_H_20_N_2_O_3_ | Feruloyl putrescine |
| **41** | 243.1720 | 14.28 | 0.751 | C_12_H_24_N_2_O_3_ | Leu-Leu |
| **42** | 245.0600 | 14.396 | 0.204 | C_9_H_14_N_2_O_4_S | Aminosalicyltaurine |
| **43** | 477.0640 | 14.959 | 0.181 | C_17_H_22_N_2_O_10_S_2_ | Neoglucobrassicin / Methoxyglucobrassicin Peak 1 |
| **44** | 470.2620 | 15.358 | 4.610 | C_27_H_43_NO | Unknown |
| **45** | 277.1560 | 16.99 | 0.493 | C_15_H_22_N_2_O_3_ | Leu-Phe |
| **46** | 754.3040 | 17.11 | 4.831 | C_39_H_49_NO_14_ | Rifamycin B |
| **47** | 585.2340 | 17.29 | 3.507 | C_24_H_49_O_9_P | Glycerophospholipid (18:0/0/0) |
| **48** | 430.2090 | 17.305 | 1.790 | C_22_H_33_N_3_O_3_ | Unknown |
| **49** | 675.2980 | 17.534 | 0.062 | C_30_H_45_NO_15_; C_37_H_44_N_4_O_3_; C_37_H_52_O_4_ | Unknown |
| **50** | 259.0760 | 17.81 | 0.470 | C_10_H_16_N_2_O_4_S | Biotin D-sulfoxide |
| **51** | 215.0500 | 17.812 | 0.911 | C_8_H_12_N_2_O_3_S | Bisnorbiotin |
| **52** | 477.0650 | 17.957 | 0.738 | C_17_H_22_N_2_O_10_S_2_ | Neoglucobrassicin / Methoxyglucobrassicin Peak 2 |
| **54** | 762.4030 | 19.371 | 1.210 | C_36_H_65_NO_13_ | Unknown |
| **55** | 1067.3800 | 19.709 | 0.344 | C_43_H_81_O_19_P_3_ | Phosphatidylinositol diphosphate (PIP2 16:0/18:2) |
| **56** | 251.0470 | 20.497 | 0.605 | C_9_H_14_N_2_O_3_S | Norbiotin (Na adduct) |
| **57** | 229.0650 | 20.505 | 3.033 | C_9_H_14_N_2_O_3_S | Norbiotin |
| **58** | 259.0760 | 20.506 | 0.441 | C_10_H_16_N_2_O_4_S | Biotin D-sulfoxide |
| **59** | 721.3300 | 20.676 | 1.326 | C_33_H_56_O_9_ | Unknown |
| **60** | 609.1460 | 21.455 | 1.010 | C_27_H_30_O_16_ | Kaempferol diglucoside/ sophoroside |
| **61** | 386.0880 | 21.638 | 4.557 | C_19_H_14_O_8_ | Unknown |
| **62** | 639.1560 | 21.873 | 0.677 | NF | Unknown |
| **63** | 279.1350 | 21.911 | 0.401 | C_15_H_24_O_2_ | Unknown |
| **64** | 512.2180 | 22.757 | 1.550 | C_23_H_42_NO_7_P | 1-18:3-lysoPE |
| **65** | 441.1810 | 23.505 | 3.506 | C_22_H_32_N_2_O_5_ | Unknown |
| **66** | 229.0650 | 23.699 | 2.983 | C_9_H_14_N_2_O_3_S | Norbiotin |
| **67** | 723.2140 | 24.08 | 0.599 | C_33_H_40_O_18_ | Feruloyl-sinapoyl-gentiobiose |
| **68** | 215.1400 | 24.884 | 0.314 | C_9_H_18_N_2_O | Unknown |
| **69** | 929.2710 | 26.816 | 2.046 | C_42_H_46_N_4_O_16_ | Unknown |
| **70** | 320.1610 | 27.408 | 1.021 | C_17_H_27_NO2 | Unknown |
| **71** | 526.2720 | 27.457 | 0.790 | C_26_H_40_N_4_O_6_ | Leu-Ile-Tyr-Pro |
| **72** | 114.0560 | 28.013 | 0.684 | C_5_H_9_NO_2_ | Unknown |

NF = no formula could be fitted; Similar groups of components are coloured coded.
